# Supplementary material for: Empirical methods for controlling false positives and estimating confidence in ChIP-Seq peaks
Source: BMC Bioinformatics. 2008 Dec 5;9:523. doi: 10.1186/1471-2105-9-523 (PMC2628906; doi:10.1186/1471-2105-9-523)
Supplement: Additional File 1 — A variety of html documents from the USeq web site detailing the available applications, their best usage, output file type descriptions, command line menus, etc. [file 1471-2105-9-523-S1.zip › USeqUserGuides/JavaDoc.zip!/JavaDoc/util/bio/annotation/package-tree.html]

util.bio.annotation Class Hierarchy


|  |  |  |
| --- | --- | --- |
| **Overview** \| **Package** \| Class \| Use \| **Tree** \| **Deprecated** \| **Index** \| **Help** | |  |
| **PREV**   **NEXT** | **FRAMES**    **NO FRAMES**     **All Classes** |  |


---


## Hierarchy For Package util.bio.annotation

**Package Hierarchies:**: All Packages

---

## Class Hierarchy

- java.lang.Object
  - util.bio.annotation.**Coordinate** (implements java.lang.Comparable<T>)
    - util.bio.annotation.**ScoredCoordinate**
    - util.bio.annotation.**ExonIntron** (implements java.lang.Comparable<T>, java.io.Serializable)- util.bio.annotation.**ExportExons**- util.bio.annotation.**ExportIntergenicRegions**- util.bio.annotation.**ExportIntronicRegions**- util.bio.annotation.**GeneGroup** (implements java.lang.Comparable<T>, java.io.Serializable)- util.bio.annotation.**GeneRep** (implements java.io.Serializable)- util.bio.annotation.**GenericFeature** (implements java.lang.Comparable<T>)- util.bio.annotation.**StartStop** (implements java.lang.Comparable<T>, java.io.Serializable)- util.bio.annotation.**Transcript** (implements java.io.Serializable)- util.bio.annotation.**TransGroup** (implements java.io.Serializable)- util.bio.annotation.**Translation** (implements java.io.Serializable)

---


|  |  |  |
| --- | --- | --- |
| **Overview** \| **Package** \| Class \| Use \| **Tree** \| **Deprecated** \| **Index** \| **Help** | |  |
| **PREV**   **NEXT** | **FRAMES**    **NO FRAMES**     **All Classes** |  |


---
